# Beyond density: Environmental and dietary drivers of endoparasite burden in overabundant red deer populations

**DOI:** 10.1007/s00436-026-08627-z

**Published:** 2026-01-27

**Authors:** Laura Fuentes-Moyano, Irene Torres-Blas, Helena Martínez-Torres, Victor Lizana, INCREMENTO Consortium, Santiago Lavín, Jordi López-Ramon, Carmen Català-Tetuán, Ramón Perea, Francisco Ruíz-Fons, Emmanuel Serrano, Jesús Cardells

**Affiliations:** 1 Wildlife Ecology & Health Group (WE&H, www.weh.cat) and Servei d’Ecopatologia de Fauna Salvatge (SEFaS), Departament de Medicina I Cirurgia Animals, Facultat de Veterinària, Universitat Autònoma de Barcelona (UAB), Bellaterra, Barcelona, Spain; 2 Servicio de Análisis, Investigación, Gestión de Animales Silvestres (SAIGAS) and Wildlife Ecology & Health group (WE&H). Facultad de Veterinaria, Universidad Cardenal Herrera-CEU, Valencia, Spain; 3 Plant & Animal Ecology Lab (PAELLA). Centro para la Conservación de la Biodiversidad y el Desarrollo Sostenible (CBDS), Universidad Politécnica de Madrid, Madrid, Spain; 4 Instituto de Investigación en Recursos Cinegéticos (IREC), CSIC-UCLM-JCCM, Ciudad Real, Spain

**Keywords:** *Cervus elaphus*, Fecal egg count, Gastrointestinal nematodes, Host density, Mediterranean

## Abstract

Red deer (*Cervus elaphus*) populations are increasing in both number and range, raising concerns that parasite abundance, such as gastrointestinal nematodes, may be influenced by host density. Research exploring this relationship in wildlife remains scarce, particularly in Mediterranean ecosystems. This field study aimed to investigate the impact of red deer overabundance on gastrointestinal nematode parasitism in two Mediterranean areas: Quintos de Mora (central Spain) and Muela de Cortes (eastern Spain). We used four enclosures, comprising two overabundance scenarios: high density (~ 45–58 deer/km²) and hyper density (~ 93–106 deer/km²). Parasite burdens were initially assessed using individual fecal samples, revealing low nematode loads across all individuals. Parasite dynamics were then monitored through pooled fecal samples over a 15–18-month period, while deer diet composition was analyzed to provide ecological context. Nematode egg excretion declined over time in all enclosures, reaching undetectable loads in three enclosures by 11 months after deer introduction, regardless of host density. Diet analyses indicated a clear dominance of woody vegetation, accounting for up to 86% of the diet in Quintos de Mora and up to 68% in Muela de Cortes, while the proportion of graminoids remained minimal. This depletion of herbaceous structure and the dominance of tannin-rich woody species containing high antiparasitic properties could contribute to reduced low parasite loads. Additional work testing the hypothesis would benefit from increased replication and higher initial worm loads. Clinical trial number: Not applicable.

**Graphical Abstract:**

**Figure:**
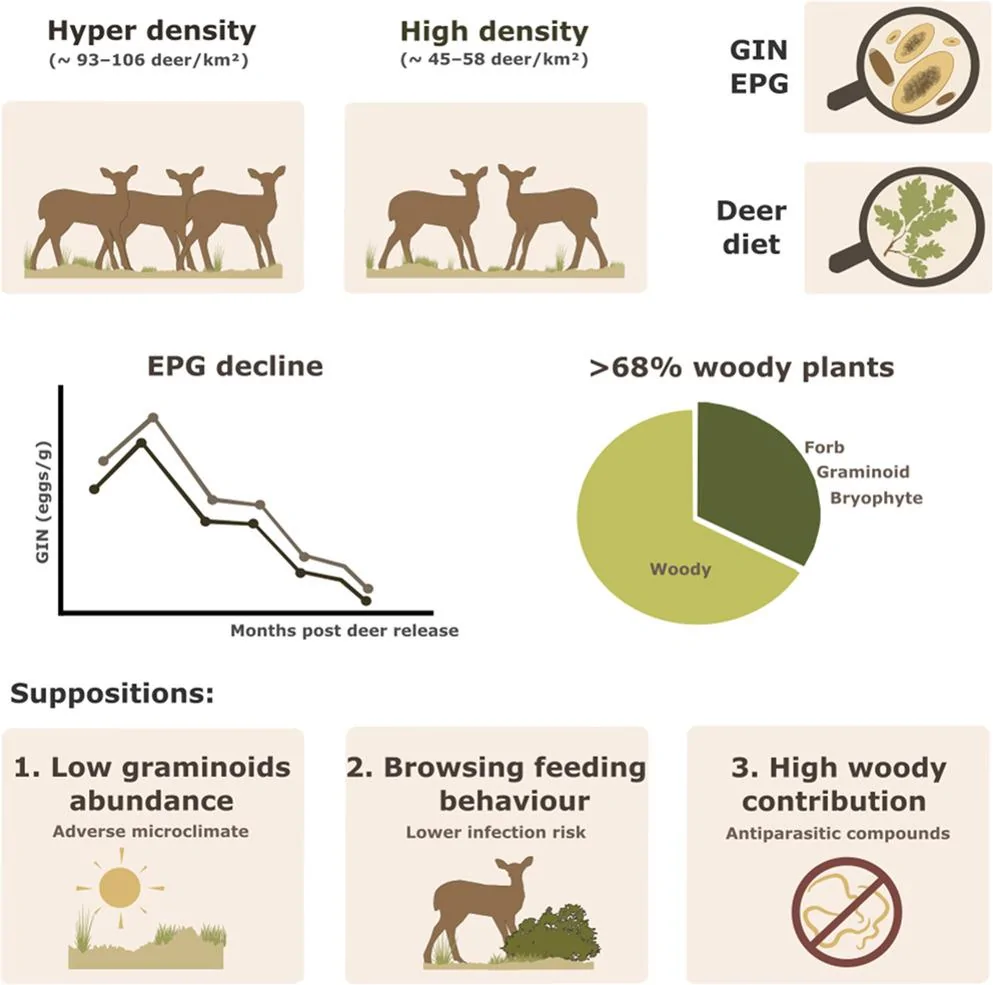

## Introduction

Ungulate populations are increasing in number and range across Europe (Milner et al. 2006; Linnell et al. 2020). For some ungulate species, such as red deer (*Cervus elaphus *L.), populations have increased markedly across most of the continent, except for the south-eastern region, rising by approximately 600,000 individuals over two decades (from the mid-1980s to the early 2000s). During this period, Spain’s red deer population increased 2.75-fold, positioning the country among those supporting the largest populations in Europe (Burbaite and Csányi 2010). This resurgence is attributed to several factors, including rural abandonment, habitat restoration and the absence of natural predators (Mattioli et al. 2022). However, human intervention has been the main contributing force to the increase in red deer populations (van Beeck Calkoen et al. 2023). In open managed hunting estates, which represent the predominant management system across Europe, supplementary feeding is frequently used to maintain high deer densities (Carpio et al. 2021). In fenced managed estates, such as those in some areas of Spain, deer are deliberately kept at very high densities for commercial hunting (up to 68 individuals km^2^; Carpio et al. 2021). Such high population densities may lead to overabundance scenarios, facilitating the persistence of pathogens relevant to livestock and human health (González-Barrio et al. 2015) and increasing the risk of disease outbreaks (Gortázar et al. 2006). Infection transmission depends on exposure to the infectious agent and the host’s susceptibility upon contact (Sweeny and Albery 2022).

Host overabundance and aggregation can increase the likelihood of pathogen transmission and persistence within a population by raising contact rates, leading to more frequent interactions among animals and, consequently, a higher basic reproduction rate of the pathogen (R_0_; Gortázar et al. 2006). Moreover, transmission is not only driven by exposure to the infectious agent but also by the host’s susceptibility upon contact (Sweeny and Albery 2022). In non-managed populations, for instance, overcrowding intensifies inter- and intraspecific competition for resources and increases nutritional and physiological stress, facilitating pathogen persistence (Lindsey et al. 2009). As a result, hosts are more exposed and susceptible to infection due to a process called the “crowding-stress hypothesis” (Steinhaus 1958). The effect of host density on infection risk can vary among parasite species and transmission strategies, with recent studies suggesting that environmentally mediated contacts may increase at high host densities, potentially enhancing transmission of parasites with indirect life stages (Albery et al. 2025). These effects of overabundance on pathogen transmission have been observed across a wide range of pathogen types, including macroparasites (e.g., nematodes, tapeworms, arthropods) and microparasites (bacteria, prions, protozoa, viruses), as shown in Table 1.

**Table 1:** Overview of studies indicating a positive relationship between host density and increased parasite abundance or pathogen transmission across different hosts and parasites

Type	Parasite species	Host	Mechanism	Reference
Ectoparasite	Tick species	Moose; bank voles and wood mice; sika deer, wild boar, raccoon dog and asiatic black bear	Vector-borne transmission	Ostfeld et al. 2001; Li et al. 2014; Krawczyk et al. 2020; Iijima et al. 2022; DeCesare et al. 2024
Endoparasite (worm)	Gastrointestinal nematode species	Mammal species; primates; black rhinoceros; european ground squirrel	Ingestion (fecal-oral route)	Arneberg et al. 1998; Arneberg 2001; Nunn et al. 2003; Santín-Durán 2004; Body et al. 2011; Stringer and Linklater 2015; Kachamakova et al. 2023
Ectoparasite	Flea species	Wagner’s gerbil	Vector-borne transmission	Krasnov et al. 2002
Bacteria	*Brucella* spp.	Elk and bison	Direct and indirect contact (excretions, fomites)	Dobson and Meagher 1996; Proffitt et al. 2015
Bacteria	*Borrelia burgdorferi* (Tick-borne disease – Lyme disease)	White-footed mice	Vector-borne transmission	Ostfeld et al. 2001
Protozoa	*Plasmodium* spp. (Vector borne disease - Malaria)	Avian species	Vector-borne transmission	Medeiros et al. 2015
Protozoa	*Leishmania* spp. (Vector borne disease)	Mammal species	Vector-borne transmission	Kocher et al. 2023
Virus	Buggy Creek Virus (Arthropod-borne virus)	Nestling house sparrows	Vector-borne transmission	O’Brien and Brown 2011
Virus	Parvovirus	African lion	Direct and indirect contact (excretions, fomites)	Packer et al. 1999
Virus	Morogoro arenavirus	Multimammate mice	Direct and indirect contact (excretions, fomites)	Mariën et al. 2020

Gastrointestinal nematodes (GIN), for example, are transmitted via the fecal-oral route, when herbivores ingest grass or water contaminated with the infective larvae (Anderson 2000). Grazing hosts minimize risk of infection by avoiding fecal contaminated areas (Coulson et al. 2018), but overcrowding limits this behavior, leading to new infections (Gulland and Fox 1992). However, some studies failed to find a positive host-parasite density relationship (Arneberg 2001; Body et al. 2011; Celva et al. 2023) likely because GIN transmission may be strongly influenced by host traits affecting susceptibility to infection (Body et al. 2011; Villalba et al. 2014; Albery et al. 2018, 2024; Hayward et al. 2022; Wiersma et al. 2023) and by abiotic factors (e.g. environmental conditions) limiting larval survival (Wilson et al. 2009; Wiersma et al. 2023).

Despite the several studies exploring these host-parasite relationships, few are conducted in Mediterranean ecosystems. In particular, the influence of red deer population density on GIN infection intensity remains poorly explored. While there are few studies investigating host traits affecting GIN prevalence (Santín-Durán et al. 2008; De La Peña et al. 2020). To our knowledge, there is only one study on host density in Spain (Santín-Durán et al. 2004), in which culled herds from different localities were examined, showing that animals from high density populations exhibited a higher parasitic burden. However, the study was observational with herds from different localities, so it did not consider the intrinsic and extrinsic factors that might influence endoparasite abundance.

The present study aimed to conduct a manipulative experiment on female red deer kept at two overabundance scenarios, high density (mean 45–58 deer/km²) and hyper density (mean 93 − 106 deer/km²) with Mediterranean vegetation, to investigate whether the density-dependence hypothesis operates for GIN burden. We predict parasite burdens to rise over time due to increased opportunities for host-parasite encounters within enclosed populations, particularly in those maintained at hyper densities enclosures. Through repeated sampling in a controlled population, this study offers a novel perspective on red deer density and parasitism, especially in the Mediterranean environment.

## Materials and methods

### Study areas

The experiment was conducted in two Mediterranean environments in central (Quintos de Mora, QM) and eastern (Muela de Cortes, MC) Spain.

**Fig. 1:**
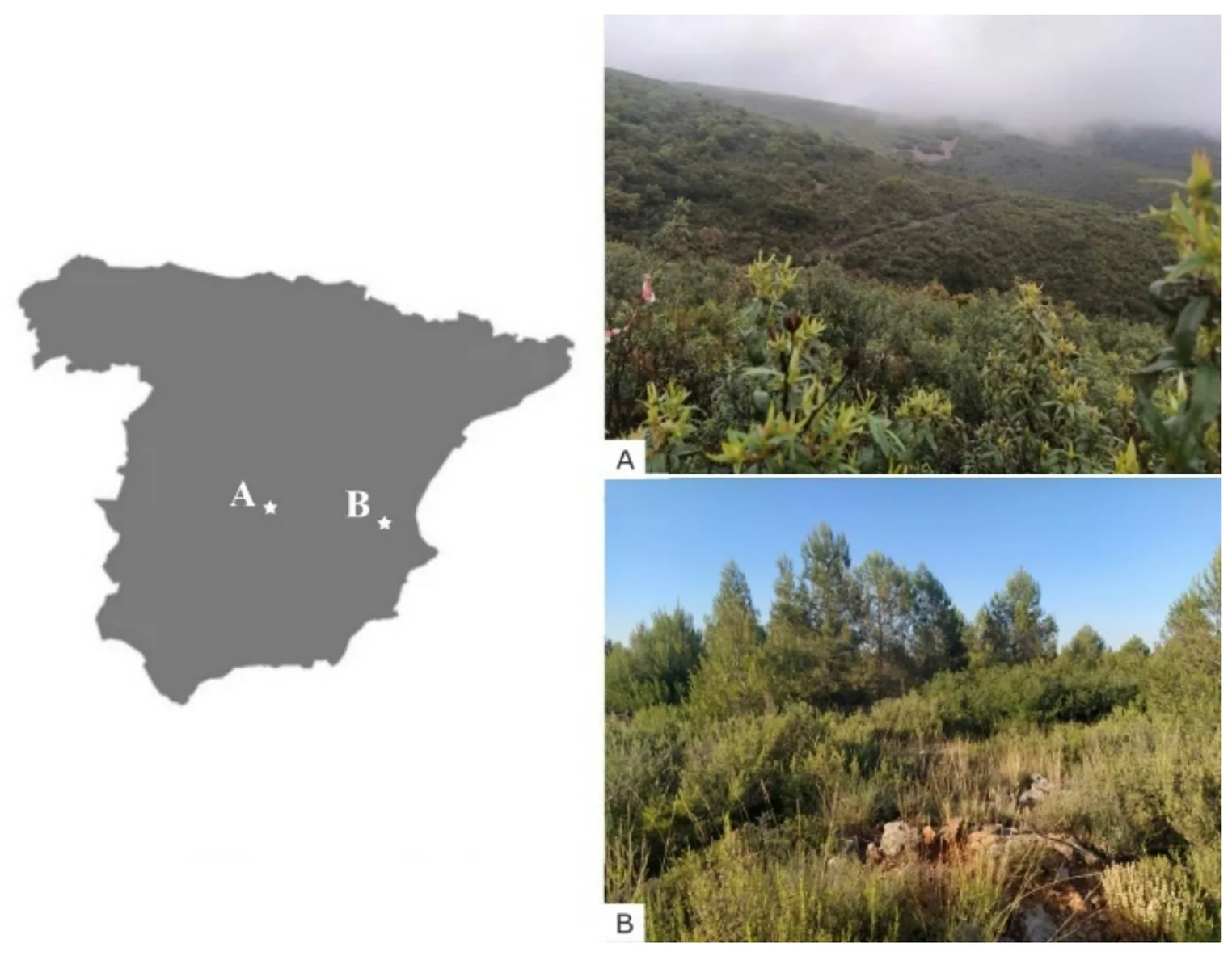
(**A**) Quintos de Mora (central Spain) and (**B**) Muela de Cortes (eastern Spain), both characterized by a Mediterranean climate, with a dominance of evergreen shrub vegetation

QM (Fig. 1a) is a 6,864-hectare public hunting estate located in the Montes de Toledo, central Spain, at an elevation ranging from 740 to 1,235 m a.s.l. (39°26’48.57"N, 4º5'57.38"W). The area has a continental Mediterranean climate, characterized by hot, dry summers lasting three to five months and cold, wet winters. The mean annual temperature is 14.7 °C, with notable seasonal variations, reaching an average of 25 °C in July and 5.7 °C in January with recurrent frost. Annual precipitation is highly variable, ranging from 200 mm to over 1,500 mm (San Miguel et al. 1997; Peláez et al. 2017). During the endoparasite study (March 2021 - June 2022; 15 months), the average summer temperature was 23.35 °C with a peak of 43.5 °C recorded on August 14, 2021. In winter, the average temperature was 7.09 °C. Total precipitation during the 15-months was 573.1 mm (data from the Quintos de Mora meteorological station).

Quartzite and slate are the predominant lithological substrates. The landscape is mainly characterised by oak woodlands (*Quercus* spp.), with a diverse shrub layer composed primarily of evergreen species from the genera *Cistus*, *Salvia*, *Rubus*, *Erica*, *Thymus*, and *Lavandula*. Patches of annual herbaceous vegetation are also present (San Miguel et al. 1997, 2013; Peláez et al. 2017; Cuerdo et al. 2025).

MC (Fig. 1b) is a 36,000-hectare game reserve located in Cortes de Pallás, at an elevation ranging from 300 to 1,000 m a.s.l. (39°12’58.3"N, 0º54’41.8"W), eastern Spain. The area has a subarid Mediterranean climate, characterized by hot, dry summers and mild winters. The mean annual temperature ranges from 14 to 16 °C, with drastic variability, averaging around 10 °C in winter and rarely dropping below 0 °C. In summer, the average temperature is 24 °C, with high diurnal temperatures reaching 31–33 °C. Annual precipitation ranges from 400 to 550 mm, typically occurring in spring and autumn (Hermosilla and Iranzo 2003; Ayuntamiento Cortes de Pallás 2016). During the endoparasite study (June 2021 - December 2022; 18 months), the average summer temperature was 25.4 °C with a peak of 45.7 °C recorded on August 14, 2022. In winter, the average temperature was 8.9 °C. Total precipitation during the 18-month period was 571 mm, according to data from the Jalance meteorological station (AEMET 2025; 373 m a.s.l., approximately 15 km from the study area).

The reserve is situated on a limestone massif with calcareous soil. Recurrent intense wildfires, along with agricultural, livestock and forestry activities, have contributed to the degradation of natural vegetation. Consequently, the landscape is dominated by an evergreen scrubland, including *Salvia rosmarinus*, *Erica multiflora*, *Ulex parviflorus*, *Thymus* spp., interspersed with patches of oak - particularly *Quercus coccifera* - and pine, mainly *Pinus halepensis*. Grassland patches are also common, especially those composed of perennial herbaceous species such as *Brachypodium retusum* and various annual species (Hermosilla and Iranzo 2003; Ayuntamiento Cortes de Pallás 2016).

### Red deer density experiment

Between February and April 2020, we rebuilt two enclosures in QM and two in MC that were built in 2000 to protect vegetation from browsing. The early enclosures were not completely effective, allowing periodical incursions of red deer, wild boar (*Sus scrofa*), roe deer (*Capreolus capreolus*) in QM and European mouflon (*Ovis musimon*) and wild boar in MC. Since our repairs in 2020, enclosures have been resistant to ungulate incursions.

Female red deer came from Cabañeros National Park (Toledo) and a nearby game ranch in central Spain. Prior to release, they spent ten months in quarantine pen. Under veterinary supervision, deer were chemically immobilized and transported to the enclosures using stretchers (Martínez-Torres et al. 2022). During handling, individuals were blindfolded, had their limbs tied, and were weighed, measured, and examined. At the start of the experiment, females had an average age of 21 months old and a mean body weight of 61 kg.

In March 2021, female red deer were released into the two enclosures of approximately 7 ha each in Quintos de Mora, with median densities of 106 individuals/km^2^ (hyper density) and 45 individuals/km^2^ (high density). In June 2021, deer were released into the two enclosures of around 10 ha each in Muela de Cortes, with median densities of 93 individuals/km^2^ (hyper density) and 58 individuals/km^2^ (high density). The high density treatment mimics the situation of many natural areas from Iberia (> 30 deer/km^2^; Vicente et al. 2007; Perea et al. 2014) central Europe (Borkowski et al. 2019) or other areas of America where red deer have been introduced (Charro et al. 2018). The hyper density treatment simulates a typical commercial and intensively-managed game estate of Central and Southern Iberia (> 90 deer/km^2^; Acevedo et al. 2008; Azorit et al. 2012). During the study period, water was available, and supplemental feeding was occasionally provided in feeding stations.

### Longitudinal sampling of gastrointestinal nematodes

During the chemical immobilization to introduce the deer into the enclosures, fecal samples were collected directly from the rectum using disposable gloves and were labelled according to the individual deer’s identification number. This procedure was carried out in March 2021 in QM and June 2021 in MC.

Fecal samples were collected following a standardized procedure. Each study area was visited every one to three months: in QM, sampling was conducted from March 2021 to June 2022, whereas in MC, samples were collected from June 2021 to December 2022. While walking inside the enclosures, separated groups of fresh feces were collected. Although individual identity could not be confirmed, samples were treated as independent for analytical purposes. Samples were placed in individually labelled plastic bags, with an average of six fecal samples collected per sampling day and per enclosure. Coprological examination was performed using a modified McMaster technique (Kassai 1999). Five grams of feces were homogenized in a mortar and mixed with 40.5 mL of water. The suspension was filtered and centrifuged at 1,500 rpm for 3 min. The supernatant was discarded, and the sediment was resuspended in flotation solution in a 10 mL tube. Each hemichamber was loaded with 0.5 mL, and the grids with 0.15 mL. Coccidia and pulmonary larvae were recorded but excluded from further analysis. Gastrointestinal nematode eggs, including *Trichuris* spp., were counted. Strongylid-type eggs, which cannot be reliably differentiated to genus level using the modified McMaster technique, were summed with *Trichuris* spp. to calculate total gastrointestinal nematode egg counts (EPG), multiplying grid counts by 33.3 and hemichamber counts by 10. Egg identification followed the guidelines of Garijo et al. (2019) and Taylor et al. (2016).

A total of 148 and 190 samples were collected from QM and MC, respectively.

### Deer’s diet characterization

Diet composition was assessed monthly over the course of one year at both sites. At QM, samples were collected from April 2021 to May 2022, and at MC from July 2021 to July 2022. For each enclosure and month, three fresh fecal samples were randomly selected for analysis. The cuticle microhistological procedure described by Espunyes et al. (2019) was followed. In this procedure, 0.5 g of each fecal sample were ground, and non-epidermal tissue was digested in concentrated HNO₃ at 80 °C for 2 min, then diluted with distilled water to stop digestion. The suspension was sieved through 1 mm and 0.25 mm meshes, and the 0.25 mm fraction was resuspended in 50% aqueous glycerin and mounted on slides. A reference epidermis collection was prepared from the plant species included in the study. Slides were examined at 400× along three 2 mm by 60 mm traverses, and up to 100 epidermal fragments per sample were identified based on cell shape, trichomes, and stomatal features. Counts were expressed as percentages per sample. For subsequent statistical analyses, plant species were classified into functional groups—woody, graminoid, forbs (non-graminoid herbaceous plants), and bryophytes—and aggregated by season.

A total of 66 fecal samples were analyzed from each study area.

### Statistical analysis

All statistical analyses and graphics were performed using RStudio 2025.09.2 + 418 (Posit Team 2025).

An initial exploration of the data was conducted following the protocol proposed by Zuur et al. (2010), which consists of examining the presence of outliers, homogeneity of variance, normality, the proportion of zeros, independence of the response variable (Y), collinearity among covariates, relationships between the response and explanatory variables, and potential interactions.

Regarding endoparasites, a few outliers were detected in the response variable but were retained. As expected in this type of data, the response variable was right skewed with a high proportion of zeros (71%), and observations were not independent due to the possibility of collecting multiple samples from the same animal within an enclosure. Initially, we used generalized additive mixed models (GAMMs), applying a log (GI + 10) transformation to the fecal egg counts to reduce overdispersion, improve the symmetry of the right-skewed distribution, and allow inclusion of zero values. This transformation enabled fitting a Gaussian error distribution while retaining all observations. To account for potential pseudoreplication among fecal samples within each enclosure, we first included enclosure ID (ID_Flock) as a random effect. However, when checking the model, we observed that the variance was almost 0 (1.7466e-05). Therefore, we decided to perform a generalized additive model (GAM) without the random factor. The smooth term for the time of sampling (Time) was modeled with a basis dimension k = −1, allowing mgcv to automatically select the effective degrees of freedom. We assumed a Gaussian error distribution with an identity link. The explanatory variables included in the models were time since red deer release (Time, in month), host density (Treatment: high and hyper densities), both predictors (Time + Treatment) and their interaction (Time × Treatment) whereas the response variable was the fecal egg counts. Two independent models were fitted, one for each study area. Model selection was based on the Akaike Information Criterion corrected for small sample sizes (AICc), and the model with the lowest AICc value was considered the most parsimonious. We used the package “mgcv” 1.9–4.9 version for this analysis (Wood 2011).

Regarding diet composition, no outliers were detected. Values were expressed as percentages (proportional data) summing to 100% across the different functional groups. To explore differences among functional groups within each season and treatment, we applied a 4-sample test for equality of proportions without continuity correction (Chi-square test). We note that, due to the compositional nature of the data, these tests provide an approximate assessment rather than a strict statistical inference. Subsequently, the same approximate test was applied to the woody category by study area to assess differences among seasons for the most frequent functional group.

## Results

### Longitudinal gastrointestinal nematodes sampling

**Fig. 2:**
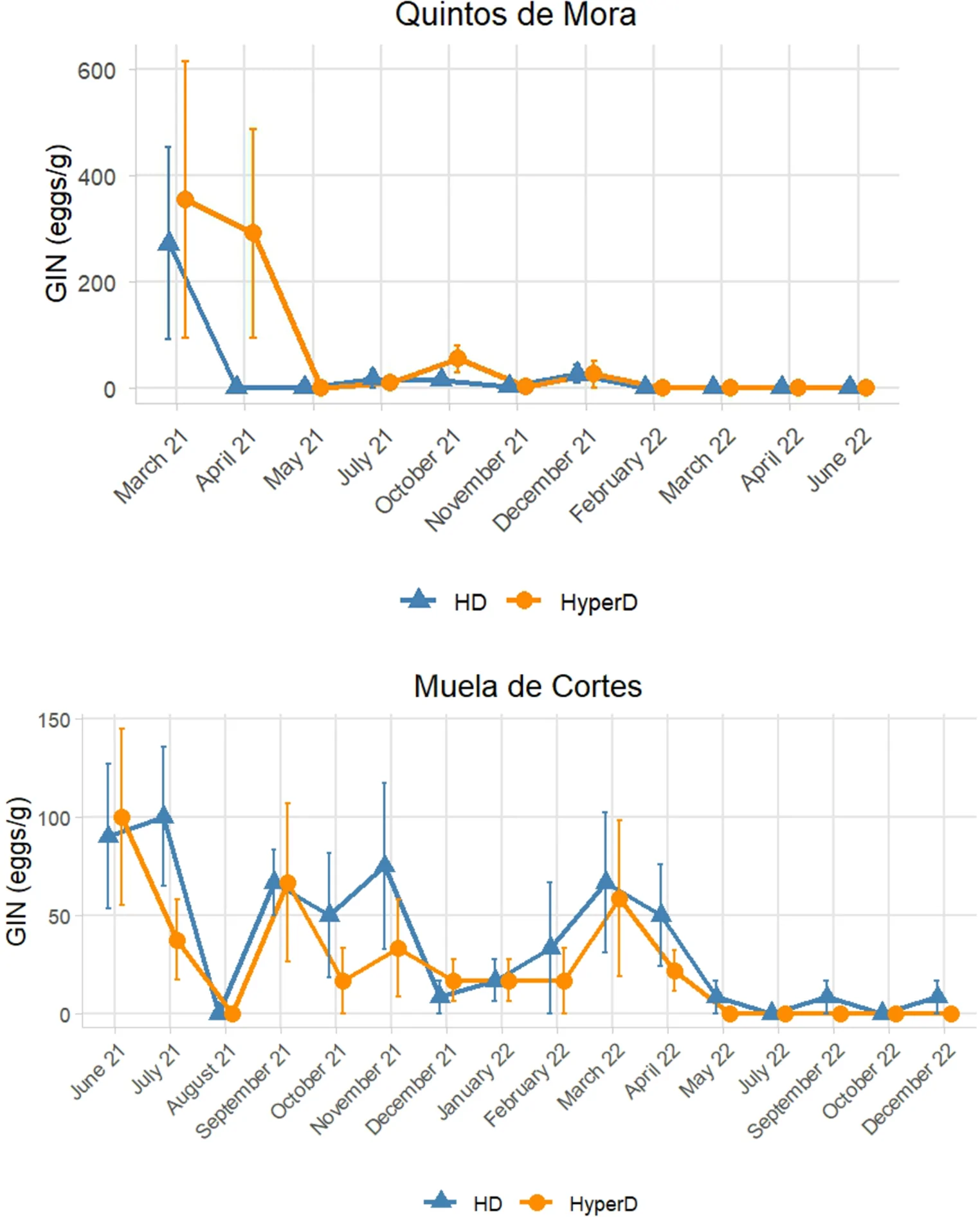
Temporal trend of the mean gastrointestinal nematode fecal counts (eggs per gram of feces) in red deer herds kept at high (HD, mean 45–58 individuals/km^2^) and hyper densities (HyperD, mean 93 −106 individuals/km^2^) in two enclosures set in two Mediterranean environments: Quintos de Mora (central Spain) and Muela de Cortes (eastern Spain). Vertical bars represent the standard error of the mean. The data were assessed from 148–190 fecal samples collected from Quintos de Mora and Muela de Cortes enclosures, respectively

A natural decline in fecal egg counts was observed over time in all enclosures (Fig. 2).

In Quintos de Mora, the initial mean GIN burden in the hyper density enclosure was 354.2 eggs/g (EPG), with one individual reaching a maximum of 3,200 EPG and two deer with undetectable egg counts (no eggs detected in a sample, reflecting the sensitivity limit of the technique, < 10 EPG). In the high density enclosure, the initial mean was 271.4 EPG, with one deer reaching 1,350 EPG (maximum) and another individual with undetectable egg counts. Eleven months after the start of the experiment, all samples from both enclosures consistently showed undetectable egg counts.

Model selection based on AIC indicated that the best-fitting model included an interaction between time since deer release and host density (see Table 2). Treatment-specific smooth terms for time were significant in both density treatments (high density: edf = 6.95, F = 5.13, *p* < 0.0001; hyper density: edf = 8.26, F = 9.57, *p* < 0.0001), predicting a marked temporal decline in fecal egg counts over the study period. Estimated marginal parasite loads did not differ significantly between treatments (*p* = 0.55, back-transformed from log (GI + 10)). The model explained 44.5% of the total variability in the data (adjusted R²). Overall, results suggest similar parasite loads between density treatments, despite differences in the shape of their temporal trajectories.

**Table 2:** Model selection for the effects of the time (months elapsed since red deer release) and red deer overabundance (Treatment: high density mean 45–58 deer/km^2^ and hyper density 93−106 deer/km^2^) on Gastrointestinal nematode fecal egg counts (eggs/gram of feces) assessed in 148 to 190 fecal samples collected in two Spanish mediterranean environments in central (Quintos de Mora) and Eastern (Muela de Cortes) Spain. The retained model is indicated in bold type

Biological Models	df	AICc	Δi_AICc	w_i_
*Quintos de Mora*				
Time * Treatment	17	404.5	0	0.92
Time + Treatment	8	410.1	5.61	0.06
Time	7	411.5	7.01	0.03
Treatment	3	470.1	65.65	0
Null model (m0)	2	472.7	68.24	0
*Muela de Cortes*				
**Time + Treatment**	10	560.2	0	0.62
Time	3	561.9	1.68	0.27
Time * Treatment	4	563.7	3.46	0.11
Treatment	2	582.8	22.62	0
Null model (m0)	1	583.1	22.85	0

*df* degrees of freedom of the model (as reported by MuMin package), *AICc * Akaike’s Information Criterion corrected for small sample sizes, *Δi* difference of AICc between each model and the most parsimonious one, *w*_*i*_ Akaike’s weight of the model

In Muela de Cortes, the initial mean GIN burden in the hyper density enclosure was 100 eggs/g, with one deer reaching 500 EPG (maximum) and four animals with undetectable egg counts. All samples consistently showed undetectable egg counts 11 months after the start of the study. In the high density enclosure, the initial mean was 90 eggs/g, with one individual reaching 200 EPG (maximum) and another with undetectable egg counts. Despite a notable reduction in egg loads over time, only one of the six final samples contained eggs (50 EPG), resulting in a final mean of 8.33 eggs/g.

In contrast, the best fitting model included additive effects of Time and Treatment (see Table 2). Time since deer release had a significant effect on parasite burden (edf = 7.01, F = 4.59, *p* < 0.001), indicating a marked temporal decline in fecal egg counts. Estimated marginal mean parasite loads were slightly higher in the high density enclosure, although this difference was not statistically significant (*p* = 0.06, back-transformed from log (GI + 10)). The model explained 15.4% of the total variability in the data (adjusted R^2^), reflecting greater temporal variability and a more complex data structure compared to QM.

### Deer’s diet characterization

In Quintos de Mora, cuticle microhistological analysis revealed that epidermal fragments of woody plants were markedly more abundant than other functional groups across all seasons and in both density enclosures. Graminoids, forbs, and bryophytes accounted for less than 10% of the diet in all cases. The proportion of woody fragments ranged from 86% to 94.8%, with no notable differences observed among seasons. Exploratory statistical tests indicated that woody plants were the most abundant functional group (X² values ranging from 265.46 to 347.16, df = 3, *p* < 0.001), with no significant seasonal differences (X² = 9.62, df = 7, *p* = 0.21).

**Fig. 3:**
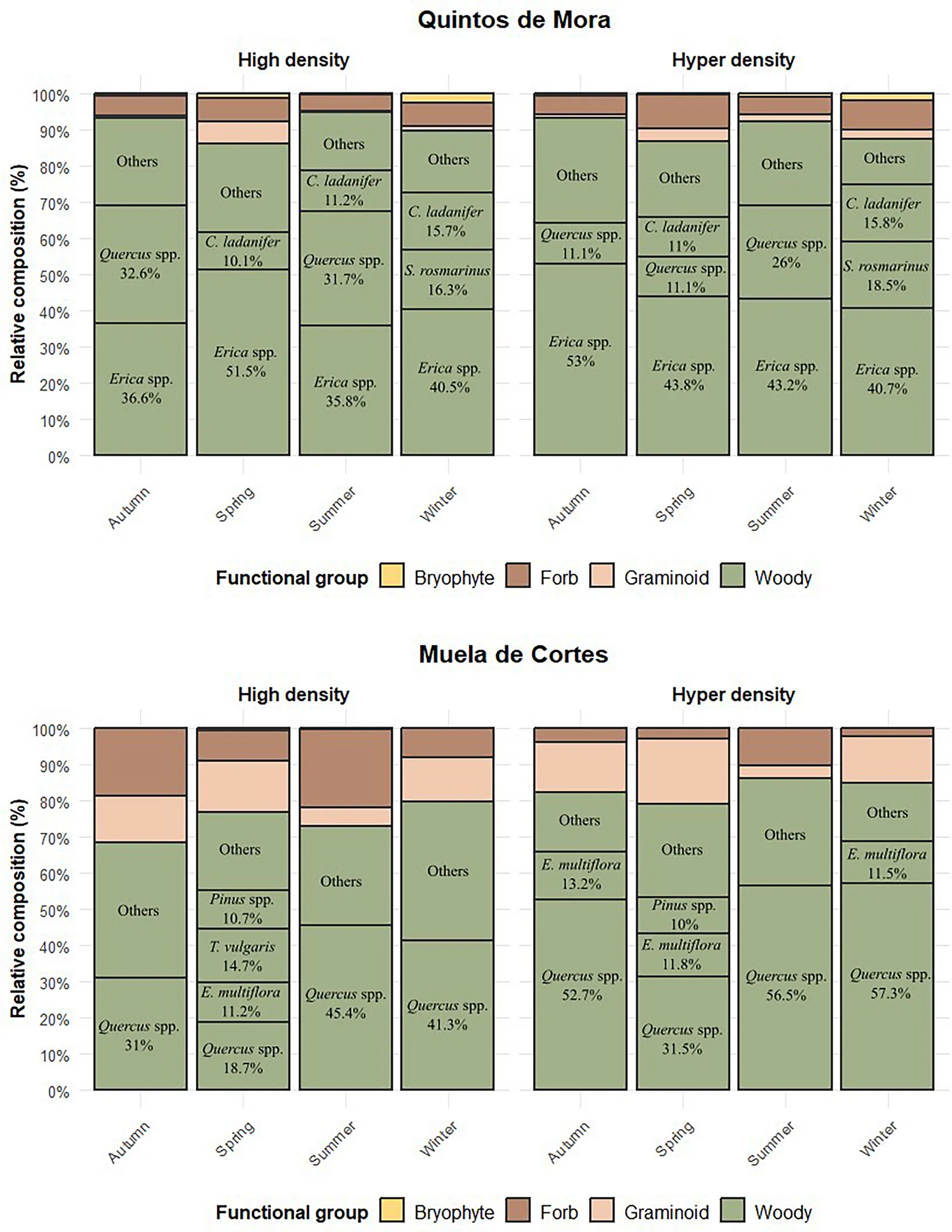
Seasonal variation in the relative composition (%) of identifiable epidermal fragments in red deer feces under overabundant conditions in two Mediterranean areas: Quintos de Mora (central Spain) and Muela de Cortes (eastern Spain). Diet composition is shown for high density (mean 45–58 deer/km²) and hyper density (mean 93–106 deer/km²) enclosures. Only plant species or genus contributing ≥ 10% to the total diet are displayed, grouped into functional categories: woody (green), graminoids (pink), forbs (brown), and bryophytes (yellow)

Although the overall contribution of woody plants remained stable, the relative proportions of individual plant species varied among seasons and showed slight differences between enclosures. Plant species accounting for more than 10% of the total diet are shown in Fig. 3. The most representative species, in decreasing order of abundance, were *Erica* spp., *Quercus* spp. (mainly *Quercus ilex*), *Cistus ladanifer*, and *Salvia rosmarinus*.

In Muela de Cortes, epidermal fragments of woody plants were also the predominant component of the diet across all seasons and in both density enclosures, although their relative contribution was lower in the high density enclosure in Autumn (68.3%) compared with the other observations. Graminoids accounted for 12.2–18%, except in summer, while forbs contributed 10.1–21.6% in autumn (only in the high density enclosure) and in summer. Bryophytes were nearly absent. Exploratory statistical tests indicated that woody plants were more abundant than other functional groups (X² values ranging from 143.06 to 269.97, df = 3, *p* < 0.001), with a significant lower proportion in the high density enclosure in autumm (X² = 15.39, df = 7, *p* = 0.03).

Similar to Quintos de Mora, the relative proportions of individual woody species varied among seasons and enclosures. The most representative species, in decreasing order of abundance, were *Quercus* spp. (mainly *Quercus ilex*), *Erica multiflora*, and *Pinus* spp.

## Discussion

Our results showed that, independently of the host density, there was a drastic reduction in the egg excretion in the overabundance scenarios.

Fecal egg counts are a valuable tool for understanding changes in parasite epidemiology (Sargison 2013) and present a positive relationship with parasite burden (Cabaret et al. 1998; Rinaldi et al. 2009), although the strength of this relationship can vary among host and parasite species and may be confounded by fluctuating egg shedding (Wood et al. 2012). Unexpectedly, and contrary to the existing literature, fecal egg counts decreased significantly in both study areas, reaching consistently undetectable burdens in both enclosures in QM and in the hyper density enclosure in MC, 11 months after the release. However, these results do not indicate a complete elimination of gastrointestinal nematode parasites or egg excretion, as the modified McMaster technique has a questionable sensibility when the EPG counts are low (Vadlejch et al. 2011; Bosco et al. 2018). Three possible explanations for this reduction are discussed in this section.

**Fig. 4:**
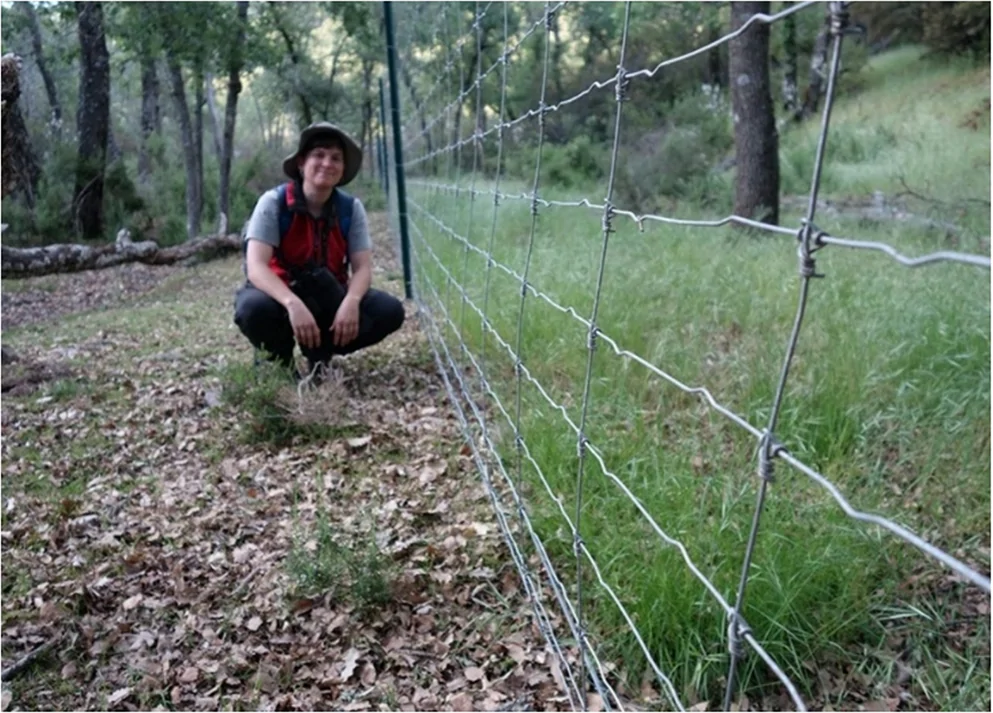
An example of complete depletion of herbaceous plants by high red deer density in Quintos de Mora (central Spain). The high density enclosure is on the left of the fence

The first one, since gastrointestinal nematodes (GIN) spend part of their life cycle outside the host, they are susceptible to environmental conditions, such as temperature (Dijk et al. 2009). The vegetation type is important to maintain the microclimate creating a suitable habitat to larvae (Wang et al. 2018). In the experimental enclosures of the present study, a recent investigation reported that increasing deer density sharply reduced herbaceous cover, richness, and diversity (Cuerdo et al. 2025). The two contrasting deer-density enclosures at Quintos de Mora, with an exclusion plot as a control, were compared across shrubland and forest habitats. Three months after deer introduction, herbaceous cover declined in both habitats, most markedly in shrubland, from 23.4% in the control to 16.1% under high density and 3.7% under hyper density. A similar decline was observed in forest (Fig. 4). This depletion of herbaceous structure may have created a more hostile microclimate for larval survival. For instance, reduced herbaceous vegetation cover may have increased the exposure to ultraviolet (UV) light, leading to raising larval mortality rates (Dijk et al. 2009), as well as reduced soil moisture, a critical factor for larval development (O’Connor et al. 2006; Khadijah et al. 2013). Besides, seasonal climatic extremes in both study areas may have further contributed to a hostile microclimate for parasite survival. In QM, recorded temperatures ranged from a minimum of − 4 °C in winter to a maximum of 43.5 °C in summer (data from the Quintos de Mora meteorological station), whereas in MC recorded temperatures ranged from a minimum of −8.2 °C in winter to 45.7 °C in summer during the study period (AEMET 2025; Jalance meteorological station).

Another possible explanation is the influence of vegetation characteristics on feeding behavior. Apio et al. (2006) found a correlation between the height of consumed plants and the probability of GIN infection, observing more infective larvae near the ground in pastures. This suggests a potentially higher infection risk for grazing animals, which feed on low-lying vegetation, compared to browsing species, which select taller plants. In our study, cuticle microhistological analysis revealed clear browsing behavior by deer, with woody plants dominating the diet. Graminoids were minimal, representing less than 10% in Quintos de Mora and up to 18% in Muela de Cortes. The predominance of woody vegetation in the diet may have reduced the likelihood of ingesting infective GIN larvae.

Similarly, the high proportion of woody species within the enclosures contain secondary metabolites with antiparasitic properties (Rochfort et al. 2008). The Ericaceae family, frequently detected in the diet samples, is rich in tannins (Harnafi et al. 2007). Notably, Frutos et al. (2002) reported that *Erica arborea* from northern Spain contained up to 302.1 g of quebracho tannin equivalents per kilogram of dry matter, indication a high concentration. Evidence from both laboratory and field studies in livestock suggest that their dietary supplementation can reduce fecal egg counts, potentially by decreasing worm fertility and/or limiting the success rate of third-stage larval establishment (Moreno-Gonzalo et al. 2012; Rodríguez-Hernández et al. 2023). Moreover, animals appear to show better resistance to infections without any apparent negative effects on their nutritional status (Moreno-Gonzalo et al. 2012). Other less frequently consumed species, for which there is published evidence of their potential anthelmintic proprieties, are *Salvia rosmarinus* (Aouadi et al. 2021), *Thymus vulgaris* (Ferreira et al. 2016) and *Pinus halepensis* (Elkady et al. 2021). Saric et al. (2015) study found no anthelmintic effect on fecal egg counts in naturally infected adult sheep after 30 days of supplementation with a mixture of high tannin-rich Mediterranean shrubs. This mixture included *Pistacia lentiscus* and *Arbutus unedo*, which was rarely consumed in our experiment, as well as *Quercus ilex*, one of the most frequently consumed species. However, comparisons with our study are limited due to differences in animal species, quantities consumed and the duration of consumption and monitoring.

Therefore, it is possible that the cumulative effect of these factors contributed to the reduction in fecal egg counts observed in the results. Furthermore, it should be emphasized that the deer exhibited very low parasitic loads at the beginning of the experiment, especially those in MC. For comparison, Jovanovic et al. (2024) studied fecal samples of 158 red deer from a Serbian hunting ground and found that most had moderate (250–800 EPG) to high (> 800 EPG) intensity infections of gastrointestinal strongyles.

In conclusion, under the conditions of our field study, red deer overabundance did not lead to an increase in gastrointestinal nematode burden. In fact, fecal egg counts decreased across all treatments, contrary to common reports in the literature. The predominance of woody plants in the diet, together with extreme environmental conditions, may have contributed to this reduction. These findings highlight the need for further field experiments to investigate the relative contributions of abiotic and biotic factors, including vegetation structure and diet composition, to endoparasite dynamics in wild ungulate populations.

## Data Availability

The experimental data and R script on gastrointestinal nematode burdens that support the findings of this study are available in the CORA.RDR repository (DOI: https://doi.org/10.34810/data2941). Data supporting the findings on red deer diet are available from the corresponding authors upon reasonable request.
